# Acknowledgment to reviewers of *Extracellular Vesicles and Circulating Nucleic Acids* in 2025

**DOI:** 10.20517/evcna.2026.12

**Published:** 2026-04-08

**Authors:** EVCNA Editorial Office

**Affiliations:** Suite 1504, Tower A, Xi’an National Digital Publishing Base, No. 996 Tiangu 7th Road, Gaoxin District, Xi’an 710077, Shaanxi, China.

In 2025, *Extracellular Vesicles and Circulating Nucleic Acids* (*EVCNA*) made significant strides, receiving its first Impact Factor of 4.8 and earning a Q1 ranking in the Biochemistry & Molecular Biology category. Additionally, *EVCNA* was indexed in the Open Access Journal Index (OAJ). These milestones were made possible through the unwavering support of the research community, whose dedication played a pivotal role in advancing *EVCNA* to this level of success. The Editorial Office of *EVCNA* extends its heartfelt gratitude to the reviewers [Table 1], who generously dedicated their time and expertise throughout the year to evaluate manuscripts. Their invaluable feedback has been essential in maintaining the quality and reputation of the journal.

**Table 1:** List of Reviewers for *EVCNA*, 2025

Ageta, Hiroshi (Japan)	Huang, Zhaohui (China)	Rai, Alin (Australia)
Akbar, Naveed (United Kingdom)	Hwang, Weilun (Taiwan, China)	Rak, Janusz W. (Canada)
Amadio, Marialaura (Italy)	Iaccino, Enrico (Italy)	Rehman, Fawad Ur (Pakistan)
Andreani, Lorenzo (Italy)	Ieva, Raffaele (France)	Ribeiro-Rodrigues, Teresa M. (Portugal)
Andriantsitohaina, Ramaroson (France)	Innes, Roger W.B. (United States)	Richards, Christopher I. (United States)
Aqil, Farrukh (United States)	Inubushi, Sachiko (Japan)	Romani, Rita (Italy)
Asa'ad, Farah (Sweden)	Ishii, Noriyuki (Japan)	Rong, Jianhui (China)
Bart, Geneviève (United Kingdom)	Jeppesen, Dennis Kjølhede (United States)	Royo, Luis J (Spain)
Baur, Andreas S. (Germany)	Jergová, Stanislava (United States)	Russo, Domenico (Italy)
Beit-Yannai, Elie (Israel)	Jia, Ruipteng Peng (China)	Sagi-Eisenberg, Ronit (Israel)
Bellezza, Ilaria (Italy)	Jiang, Peiyong (Hong Kong)	Saint-Pol, Julien (France)
Berezin, Alexander E. (Austria)	Jingushi, Kentaro (Japan)	Santiago, Ana Raquel (Portugal)
Bhattacharya, Sanjoy K. (United States)	Jones, Melissa K. (United States)	Schaack, Beatrice (France)
Bilousova, Tina (United States)	Kanada, Masamitsu (United States)	Sébire, Guillaume (Canada)
Brachner, Andreas (Austria)	Kim, Han Sang (South Korea)	Senthil, Maheswari (United States)
Bruno, Stefania (Italy)	Kim, Insan (South Korea)	Seo, Naohiro (Japan)
Buch, Shilpa (United States)	Kodali, Maheedhar (United States)	Shah, Kiran (Australia)
Buchet, Rene (France)	Kornek, Miroslaw T. (Germany)	Shang, Anquan (China)
Bussolati, Benedetta (Italy)	Kou, Xiaoxing (China)	Sheng, Kangliang (China)
Cai, Zhijian (China)	Kouroupis, Dimitrios (United States)	Shi, Yejiao (China)
Cao, Yong (China)	LaFramboise, William A. (United States)	Shiba, Kiyotaka (Japan)
Carvalho, Ana Sofia (Portugal)	Lange-Consiglio, Anna (Italy)	Shiddiky, Muhammad JA (Australia)
Casado-Diaz, Antonio (Spain)	Languino, Lucia (United States)	Shu, Bowen (China)
Chahwan, Richard (Switzerland)	Lauritzen, Inger (France)	Singh, Seema (United States)
Chalayer, Émilie (France)	Lee, Mi-young (South Korea)	Sourisseau, Marion (United States)
Chen, Chaoxiang (China)	Lehr, Claus Michael (Germany)	Stassen, Frank R.M. (Netherlands)
Cheng, Fang (China)	Lei, Jianyong (China)	Stewart, Christopher J (United Kingdom)
Chi, Zailong (China)	Lewis, Stephen M. (Canada)	Stice, Steven L. (United States)
Cho, Jongki (South Korea)	Li, Bo (China)	Suades, Rosa (Spain)
Cho, Young-Eun (South Korea)	Li, Furong (China)	Sugaya, Kiminobu (United States)
Christian, Sherri L. (Canada)	Li, Juanjuan (China)	Sui, Bingdong (China)
Conti, Pio (Italy)	Li, Min (United States)	Sun, Mingkuan (China)
Cooks, Tomer (Israel)	Li, Peng (China)	Sun, Yanyan (China)
Corrado, Chiara (Italy)	Li, Yan (United States)	Szczesny, Bartosz (United States)
Crescitelli, Rossella (Sweden)	Lian, Qizhou (China)	Teh, Bin Tean (Singapore)
D'Agostino, Vito Giuseppe (Italy)	Liang, Gaofeng (China)	Tian, Haibin (China)
Dávalos, Alberto (Spain)	Liang, Hongwei (China)	Tikhanovich, Irina S. (United States)
De la Cuesta, Fernando (Spain)	Liang, Xiuming (Sweden)	Tossetta, Giovanni (Italy)
Deng, Chunhui (China)	Liguori, Giovanna L. (Italy)	Traina, Giovanna (Italy)
Deng, Zaian (China)	Liu, Guozhen (China)	Ugaz, Victor M. (United States)
Dentice, Monica (Italy)	Liu, Han (China)	Umar, Shahid (United States)
Derderian, Sarkis Christopher (United States)	Liu, Hengrui (United Kingdom)	Urbanelli, Lorena (Italy)
Ding, Lin (China)	Liu, Jiyong (China)	Valés-Gómez, Mar (Spain)
Ding, Xin (China)	Liu, Lei (China)	Verboven, Kenneth (Belgium)
Ding, Yi-nan (China)	Liu, Liangle (China)	Verderio, Claudia (Italy)
Ekström, Karin M. (Sweden)	Liu, Qiong (China)	Vitale, Nicolas (France)
Fais, Stefano (Italy)	Lousa, Carine De Marcos (United Kingdom)	von Strandmann, Elke Pogge (Germany)
Falasca, Marcoa (Italy)	Lu, Rong (China)	Wan, Meihua (China)
Falcon-Perez, Juan Manuel (Spain)	Lucien, Fabrice (United States)	Wan, Yuan (United States)
Fan, Enguo (China)	Lundy, David J (Taiwan, China)	Wang, Chao (China)
Fan, Wei (China)	Luo, Lan (China)	Wang, Kaiyuan (China)
Fan, Zhijin (China)	Luo, Zhiwen (China)	Wang, Yongxiang (China)
Fattorossi, Andréa (Italy)	Lv, Xiongwen (China)	Weiss, Tamara (Austria)
Ferraro, Davide (Italy)	Mahida, Rahul (United Kingdom)	Weng, Weizong (China)
Fradin, Delphine (France)	Mahshid, Sara (Canada)	Whiteside, Theresa L. (United States)
Franquesa, Marcella (Spain)	Malnou, Cécile Evelyne (France)	Wong, Siu Hong Dexter (China)
FUJIHARA, Junko (Japan)	Mao, Wenjun (China)	Xia, Xiaobo (China)
Fusco, Salvatore (Italy)	Marchetti, Laura (Italy)	Xiao, Bo (China)
Gabrielsson, Susanne (Sweden)	Markovic, Svetomir N. (United States)	Xie, Junhua (China)
Galipeau, Jacques (Canada)	Martins-Marques, Tânia (Portugal)	Xu, Qingqiang (China)
Gallart-Palau, Xavier (Spain)	Melzig, Mathias Friedrich (Germany)	Xu, Wentao (China)
Galtung, Hilde Kanli (Norway)	Ménasché, Philippe (France)	Yamamoto, Yusuke (Japan)
Gao, Lie (United States)	Menck, Kerstin (Germany)	Yan, Wenjuan (China)
García, Juan Luis (Spain)	Miceli, Vitale (Italy)	Yáñez-Mó, María (Spain)
Ghatak, Subhadip (United States)	Mohanakumar, Thalachallour (United States)	Yang, Jinho (South Korea)
Giannecchini, simone (Italy)	Morasso, Carlo Francesco (Italy)	Yang, Zhen (China)
Gonzalez, Esperanza (Spain)	Morelli, Adrián E. (United States)	Yang, Zhengyang (China)
Gonzalez-Kozlova, Edgar E. (United States)	Mou, Xiaozhou (China)	Yoon, Daesung (South Korea)
Grande, Daniel A. (United States)	Neri, Tommaso (Italy)	Yoshioka, Yusuke (Japan)
Grandi, Guido (Italy)	Niu, Li‐na (China)	Yu, Fabiao (China)
Granholm, Ann Charlotte (United States)	Noren Hooten, Nicole (United States)	Yuyama, Kohei (Japan)
Grässel, Susanne (Germany)	Ouyang, Yingshi (United States)	Zannella, Carla (Italy)
Greening, David W (Australia)	Parisse, Pietro (Italy)	Zhang, Donghong (United States)
Gualerzi, Alice (Italy)	Park, Dong-Jun (United States)	Zhang, Hao (China)
Guo, Shan (China)	Park, Ki Soo (South Korea)	Zhang, Lin (China)
Hakami, Ramin Mollaaghababa (United States)	Peake, Nicholas J. (United Kingdom)	Zhang, Mingzhen (China)
Han, Guanghong (China)	Peng, Lihua (China)	Zhang, Peng (China)
Han, Pingping (Australia)	Peng, Xueqiang (China)	Zhang, Wei (United States)
Handberg, Aase (Denmark)	Piomboni, Paola (Italy)	Zhang, Xudong (China)
He, Bangshun (China)	Poetsch, Ansgar (United Kingdom)	Zhang, Yang (China)
He, Xiaoning (China)	Poirot, Marc (France)	Zhang, Ye (China)
Holliday, Lexie Shannon (United States)	Pozzobon, Michela (Italy)	Zhao, Xiangxuan (China)
Holliday, Shannon (United States)	Prados-Rosales, Rafael (Spain)	Zheng, Aiping (China)
Hsu, Chou Yi (Taiwan, China)	Qadri, Shahnaz M. (United States)	Zheng, Lei (China)
Hsu, Wei-Hsuan (Taiwan, China)	Qiao, Hongzhi (China)	Zhou, Jiajing (China)
Hu, Chenghu (China)	Qin, Xiang (China)	Zhou, Nan (China)
Hu, Liang (China)	Radhakrishna, Uppala (United States)	Zhu, Yazhen (United States)
Hu, Lianghai (China)	Ragni, Enrico (Italy)	Zöller, Margot (China)
Ageta, Hiroshi (Japan)	Huang, Zhaohui (China)	Rai, Alin (Australia)
Akbar, Naveed (United Kingdom)	Hwang, Weilun (Taiwan, China)	Rak, Janusz W. (Canada)
Amadio, Marialaura (Italy)	Iaccino, Enrico (Italy)	Rehman, Fawad Ur (Pakistan)
Andreani, Lorenzo (Italy)	Ieva, Raffaele (France)	Ribeiro-Rodrigues, Teresa M. (Portugal)
Andriantsitohaina, Ramaroson (France)	Innes, Roger W.B. (United States)	Richards, Christopher I. (United States)
Aqil, Farrukh (United States)	Inubushi, Sachiko (Japan)	Romani, Rita (Italy)
Asa'ad, Farah (Sweden)	Ishii, Noriyuki (Japan)	Rong, Jianhui (China)
Bart, Geneviève (United Kingdom)	Jeppesen, Dennis Kjølhede (United States)	Royo, Luis J (Spain)
Baur, Andreas S. (Germany)	Jergová, Stanislava (United States)	Russo, Domenico (Italy)
Beit-Yannai, Elie (Israel)	Jia, Ruipteng Peng (China)	Sagi-Eisenberg, Ronit (Israel)
Bellezza, Ilaria (Italy)	Jiang, Peiyong (Hong Kong)	Saint-Pol, Julien (France)
Berezin, Alexander E. (Austria)	Jingushi, Kentaro (Japan)	Santiago, Ana Raquel (Portugal)
Bhattacharya, Sanjoy K. (United States)	Jones, Melissa K. (United States)	Schaack, Beatrice (France)
Bilousova, Tina (United States)	Kanada, Masamitsu (United States)	Sébire, Guillaume (Canada)
Brachner, Andreas (Austria)	Kim, Han Sang (South Korea)	Senthil, Maheswari (United States)
Bruno, Stefania (Italy)	Kim, Insan (South Korea)	Seo, Naohiro (Japan)
Buch, Shilpa (United States)	Kodali, Maheedhar (United States)	Shah, Kiran (Australia)
Buchet, Rene (France)	Kornek, Miroslaw T. (Germany)	Shang, Anquan (China)
Bussolati, Benedetta (Italy)	Kou, Xiaoxing (China)	Sheng, Kangliang (China)
Cai, Zhijian (China)	Kouroupis, Dimitrios (United States)	Shi, Yejiao (China)
Cao, Yong (China)	LaFramboise, William A. (United States)	Shiba, Kiyotaka (Japan)
Carvalho, Ana Sofia (Portugal)	Lange-Consiglio, Anna (Italy)	Shiddiky, Muhammad JA (Australia)
Casado-Diaz, Antonio (Spain)	Languino, Lucia (United States)	Shu, Bowen (China)
Chahwan, Richard (Switzerland)	Lauritzen, Inger (France)	Singh, Seema (United States)
Chalayer, Émilie (France)	Lee, Mi-young (South Korea)	Sourisseau, Marion (United States)
Chen, Chaoxiang (China)	Lehr, Claus Michael (Germany)	Stassen, Frank R.M. (Netherlands)
Cheng, Fang (China)	Lei, Jianyong (China)	Stewart, Christopher J (United Kingdom)
Chi, Zailong (China)	Lewis, Stephen M. (Canada)	Stice, Steven L. (United States)
Cho, Jongki (South Korea)	Li, Bo (China)	Suades, Rosa (Spain)
Cho, Young-Eun (South Korea)	Li, Furong (China)	Sugaya, Kiminobu (United States)
Christian, Sherri L. (Canada)	Li, Juanjuan (China)	Sui, Bingdong (China)
Conti, Pio (Italy)	Li, Min (United States)	Sun, Mingkuan (China)
Cooks, Tomer (Israel)	Li, Peng (China)	Sun, Yanyan (China)
Corrado, Chiara (Italy)	Li, Yan (United States)	Szczesny, Bartosz (United States)
Crescitelli, Rossella (Sweden)	Lian, Qizhou (China)	Teh, Bin Tean (Singapore)
D'Agostino, Vito Giuseppe (Italy)	Liang, Gaofeng (China)	Tian, Haibin (China)
Dávalos, Alberto (Spain)	Liang, Hongwei (China)	Tikhanovich, Irina S. (United States)
De la Cuesta, Fernando (Spain)	Liang, Xiuming (Sweden)	Tossetta, Giovanni (Italy)
Deng, Chunhui (China)	Liguori, Giovanna L. (Italy)	Traina, Giovanna (Italy)
Deng, Zaian (China)	Liu, Guozhen (China)	Ugaz, Victor M. (United States)
Dentice, Monica (Italy)	Liu, Han (China)	Umar, Shahid (United States)
Derderian, Sarkis Christopher (United States)	Liu, Hengrui (United Kingdom)	Urbanelli, Lorena (Italy)
Ding, Lin (China)	Liu, Jiyong (China)	Valés-Gómez, Mar (Spain)
Ding, Xin (China)	Liu, Lei (China)	Verboven, Kenneth (Belgium)
Ding, Yi-nan (China)	Liu, Liangle (China)	Verderio, Claudia (Italy)
Ekström, Karin M. (Sweden)	Liu, Qiong (China)	Vitale, Nicolas (France)
Fais, Stefano (Italy)	Lousa, Carine De Marcos (United Kingdom)	von Strandmann, Elke Pogge (Germany)
Falasca, Marcoa (Italy)	Lu, Rong (China)	Wan, Meihua (China)
Falcon-Perez, Juan Manuel (Spain)	Lucien, Fabrice (United States)	Wan, Yuan (United States)
Fan, Enguo (China)	Lundy, David J (Taiwan, China)	Wang, Chao (China)
Fan, Wei (China)	Luo, Lan (China)	Wang, Kaiyuan (China)
Fan, Zhijin (China)	Luo, Zhiwen (China)	Wang, Yongxiang (China)
Fattorossi, Andréa (Italy)	Lv, Xiongwen (China)	Weiss, Tamara (Austria)
Ferraro, Davide (Italy)	Mahida, Rahul (United Kingdom)	Weng, Weizong (China)
Fradin, Delphine (France)	Mahshid, Sara (Canada)	Whiteside, Theresa L. (United States)
Franquesa, Marcella (Spain)	Malnou, Cécile Evelyne (France)	Wong, Siu Hong Dexter (China)
FUJIHARA, Junko (Japan)	Mao, Wenjun (China)	Xia, Xiaobo (China)
Fusco, Salvatore (Italy)	Marchetti, Laura (Italy)	Xiao, Bo (China)
Gabrielsson, Susanne (Sweden)	Markovic, Svetomir N. (United States)	Xie, Junhua (China)
Galipeau, Jacques (Canada)	Martins-Marques, Tânia (Portugal)	Xu, Qingqiang (China)
Gallart-Palau, Xavier (Spain)	Melzig, Mathias Friedrich (Germany)	Xu, Wentao (China)
Galtung, Hilde Kanli (Norway)	Ménasché, Philippe (France)	Yamamoto, Yusuke (Japan)
Gao, Lie (United States)	Menck, Kerstin (Germany)	Yan, Wenjuan (China)
García, Juan Luis (Spain)	Miceli, Vitale (Italy)	Yáñez-Mó, María (Spain)
Ghatak, Subhadip (United States)	Mohanakumar, Thalachallour (United States)	Yang, Jinho (South Korea)
Giannecchini, simone (Italy)	Morasso, Carlo Francesco (Italy)	Yang, Zhen (China)
Gonzalez, Esperanza (Spain)	Morelli, Adrián E. (United States)	Yang, Zhengyang (China)
Gonzalez-Kozlova, Edgar E. (United States)	Mou, Xiaozhou (China)	Yoon, Daesung (South Korea)
Grande, Daniel A. (United States)	Neri, Tommaso (Italy)	Yoshioka, Yusuke (Japan)
Grandi, Guido (Italy)	Niu, Li‐na (China)	Yu, Fabiao (China)
Granholm, Ann Charlotte (United States)	Noren Hooten, Nicole (United States)	Yuyama, Kohei (Japan)
Grässel, Susanne (Germany)	Ouyang, Yingshi (United States)	Zannella, Carla (Italy)

*EVCNA*: *Extracellular Vesicles and Circulating Nucleic Acids*.

Our expert reviewers are instrumental in the editorial decisions at *EVCNA*, and we are deeply appreciative of their invaluable contributions. In 2025, we introduced reviewer accounts in the submission system as a gesture of appreciation. These accounts allow reviewers to access their past reviews easily, view feedback from other reviewers, and download acknowledgment letters for their records. Additionally, we provide reviewers with APC discount vouchers to support their manuscript submissions.

To honor those who have gone above and beyond, the Editorial Office has selected two reviewers as the “2025 Best Reviewers.”

We cannot thank our reviewers enough for their generosity and dedication throughout the year. We look forward to continuing our collaboration in 2026, ensuring the peer review process remains rigorous and the high publishing standards of *EVCNA* continue to thrive!

## DECLARATIONS

### Copyright

©The Author(s) 2026.

